# A high-throughput, polymerase-targeted RT-PCR for broad detection of mammalian filoviruses

**DOI:** 10.1128/spectrum.01010-24

**Published:** 2024-07-24

**Authors:** Na Cui, Yael L. Perez, Adam J. Hume, B. Ethan Nunley, Kevin Kong, Margaret G. Mills, Hong Xie, Alexander L. Greninger

**Affiliations:** 1 Department of Laboratory Medicine and Pathology, University of Washington School of Medicine, Seattle, Washington, USA; 2 Department of Microbiology/National Emerging Infectious Diseases Laboratories, Chobanian & Avedisian School of Medicine, Boston University, Boston, Massachusetts, USA; 3 Vaccine and Infectious Disease Division, Fred Hutchinson Cancer Research Center, Seattle, Washington, USA; Erasmus MC, Rotterdam, Netherlands

**Keywords:** filovirus, RT-PCR, broad range PCR, pan-filovirus, zoonosis

## Abstract

Filoviruses are some of the most lethal viruses in the modern world, and increasing numbers of filovirus species and genera have been discovered in recent years. Despite the potential severity of filovirus outbreaks in the human population, comparably few sensitive pan-filovirus RT-PCR assays have been described that might facilitate early detection and prevention. Here, we present a new pan-filovirus RT-PCR assay targeting the L polymerase gene for detection of all known mammalian filoviruses. We demonstrate the detection of 10 synthetic filovirus RNA templates with analytical sensitivity ranging from 178 to 3,354 copies/mL, without cross-reactivity on 10 non-filoviral human viral species. We verified assay performance on 10 inactivated filovirus isolates, yielding initial sensitivities of 0.012–44.17 TCID_50_/mL. We coupled this broadly reactive RT-PCR with a deep sequencing workflow that is amenable to high-throughput pooling to maximize detection and discovery potential. In summary, this pan-filovirus RT-PCR assay targets the most conserved filovirus gene, offers the widest breadth of coverage to date, and may help in the detection and discovery of novel filoviruses.

**IMPORTANCE:**

Filoviruses remain some of the most mysterious viruses known to the world, with extremely high lethality rates and significant pandemic potential. Yet comparably few filovirus species and genera have been discovered to date and questions surround the definitive host species for zoonotic infections. Here, we describe a novel broadly reactive RT-PCR assay targeting the conserved L polymerase gene for high-throughput screening for filoviruses in a variety of clinical and environmental specimens. We demonstrate the assay can detect all known mammalian filoviruses and determine the sensitivity and specificity of the assay on synthetic RNA sequences, inactivated filovirus isolates, and non-filoviral species.

## INTRODUCTION

Filovirus-associated hemorrhagic fever is one of the most lethal diseases in the modern world, causing average fatality from 53.8% for Marburg virus to 65% for Ebola virus (1). Classified as WHO Risk Group 4 agents, filoviruses are associated with a high mortality rate, person-to-person transmission, potential to spread via aerosols, and a comparative dearth of available vaccines and therapeutic antivirals (2). Since the first discovery in 1967, filoviruses have caused over 40 outbreaks worldwide, mainly in sub-Saharan Africa. The largest outbreak to date occurred in West Africa from 2013 to 2016, involving rural and urban areas in multiple countries, with over 28,000 infections and 11,000 deaths (3). Filovirus outbreaks have generally been located in African countries, with outbreaks and secondary cases also reported in Europe, North America, and Asia (4).

In the 2023 taxonomic report of the International Committee on the Taxonomy of Viruses, *Filoviridae* expanded to include a new genus, bringing the total to nine (5). The *Loebevirus, Oblavirus*, *Striavirus*, *Tapjovirus*, and *Thamnovirus* genera are non-mammalian viral genera, discovered in fish and snakes. The other four genera, *Cuevavirus*, *Dianlovirus*, *Orthomarburgvirus*, and *Orthoebolavirus*, are associated with mammals.

Filovirus infections in humans are thought to originate as spillovers from wildlife reservoirs, leading to human-to-human transmission (4). While the natural reservoirs of filoviruses have not been fully identified, bats are the most likely candidate reservoir. Ebola virus RNA has been discovered in three naturally infected fruit bat species (6), and antibodies against the different ebolavirus species have been detected in at least 14 bat species, including 9 species that had antibodies against Ebola virus (EBOV) (7). Marburg virus, another infectious member of the filovirus family, has been isolated from Egyptian rousettes (*Rousettus aegyptiacus*) (8). More recently discovered filoviruses such as Lloviu virus, Bombali virus, and Dehong virus have been detected in Schreiber’s bats (*Miniopterus schreibersii*) (9), free-tailed bats (*Chaerephonpumilus* and *Mops condylurus*) (9), and fruit bats (*Rousettus leschenaultii*), respectively (10). Although bats are implicated as principal drivers of filovirus transmission to humans, other wildlife or livestock animal species might be involved, including pigs (11), duikers (12), dogs (13), and non-human primates (14). As virus spillover can occur at any time, a timely, accurate, and sensitive RT-PCR assay for broad detection of mammalian filoviruses in diverse human, animal, and environmental specimens is crucial for reducing filovirus disease outbreaks and discriminating from other viral hemorrhagic fever etiological agents.

To date, several RT-PCR assays for detecting ebolaviruses have been published and applied, either in-house or commercially (15). Most of these assays are based on the nucleoprotein (NP) gene, glycoprotein (GP) gene, or L gene and can detect a limited number of filovirus species (16–19). The first pan-filovirus assays were designed more than a decade ago (20, 21) and may lack coverage for newly discovered filovirus species. Other pan-filovirus assays included a broader range of species but had complicated primer sets and multiple PCR steps (22, 23). Here, we report the development of a single-reaction RT-qPCR assay targeting a region of the RNA-dependent RNA polymerase (L) gene followed by deep sequencing of resulting amplicons to enable the detection of all known mammalian filoviruses and potentially new species.

## MATERIALS AND METHODS

### Primer design

Reference sequences from eight genera and unclassified *Filoviridae* were downloaded from NCBI Taxonomy on 6 December 2022 using “Filoviridae” as search keyword. To include more sequence information, we downloaded all 37 complete or partial L gene CDS sequences on “Display level 3” and selected one for each strain/isolate from NCBI Nucleotide as representative sequence. L gene sequence of *Loebevirus percae* from a new genus Loebevirus was subsequently downloaded on 4 June 2024. Multiple alignment and phylogenetic tree were generated using Clustal Omega v1.2.3 with default parameters and FastTree v2.1.11 with default parameters. Based on sequence divergence, we focused the alignment on 28 mammalian filovirus L gene sequences (File S1). Highly conserved regions of these sequences were manually selected for designing degenerate forward and reverse primers. We replaced “N” (representing A, G, C, or T) with inosine (I) to reduce overall primer degeneracy. The final forward primer PfiloL_F 5′- CAYCARGCITCITGGCA-3′ (positions 1819–1835 of AY354458.1) and reverse primer PfiloL_R 5′- CAYTGRTTRTCHCCCATIAC-3′ (positions 2215–2234 of AY354458.1) were designed to yield a product size of 416 bp. All primers were synthesized by Integrated DNA Technologies (IDT, Coralville, IA, USA).

### *In silico* accuracy test

Filovirus L and NP gene sequences were downloaded from NCBI Nucleotide (https://www.ncbi.nlm.nih.gov/nuccore/?term=txid11266[Organism:exp]) on 3 August 2023. Sequences were filtered with SeqKit (https://github.com/shenwei356/seqkit/) to remove short lengths (NP length <2,000 bp; L length <700 bp), and sequences with ambiguous/missing nucleotides (Ns). After filtering, 3,051 L (including all reference sequences for primer design) and 3,183 NP gene sequences with length longer than 2,000 and 700 bp, respectively, were kept for *in silico* primer binding testing (Table S1). The digital *in silico* test was performed using the Test with Saved Primers option in Geneious v2023.2.1. Previously published pan-filovirus primers (21, 23, 24) were also used as input for the same primer test.

### Stool, serum, and clinical sample preparation

Incidental cat and horse stool were collected from the Puget Sound area and mixed with each other 1:1 by weight. 0.1 g stool mixture was dissolved in 1 mL 1× DNA/RNA Shield (Zymo Research, Irvine, CA, USA). The mixture was centrifuged at 7,000 × *g* for 10 min and supernatant was collected as stool matrix. De-identified remnant human serum and respiratory clinical specimens (Table S2) were collected from the UW Department of Laboratory Medicine and Pathology and pooled. This work was approved by the University of Washington Institutional Review Board under a consent waiver and determined to be exempt from IACUC review. Nipah virus RNA from Malaysia and Bangladesh strains used for specificity testing was kindly provided by Dr. Alexander Freiberg from the University of Texas Medical Branch.

### Standard template synthesis

Ten gBlocks covering four mammalian genera were designed based on the 28 reference filovirus L gene sequences and synthesized by IDT. Each gBlock is ~1,000 bp long covering the primer region, with a T7 promoter sequence added to the 5′ end to perform *in vitro* transcription (File S2). All gBlocks were synthesized by Integrated DNA Technologies (IDT, Coralville, IA, USA). *In vitro* transcribed (IVT) RNA was synthesized with MEGAscript T7 Transcription Kit (Invitrogen, Carlsbad, CA, USA). About 150 ng gBlocks dsDNA in 8 µL nuclease-free water as template was added to a mixture of 2 µL ATP, 2 µL CTP, 2 µL GTP, 2 µL UTP, 2 µL 10× Reaction Buffer, 2 µL Enzyme Mix to a standard 20 µL reaction. After incubation at 37°C for 4 h, 1 µL TURBO DNase was added and incubated at 37°C for 15 min to digest DNA. The IVT RNA was mixed with 30 µL nuclease-free water and 30 µL LiCl Precipitation solution, chilling for 1 h at −20°C. After centrifuging at 4°C, 16,000 × *g*, the pellet was washed with 70% ethanol and resuspended in nuclease-free water. The A280 of IVT RNA was measured by NanoDrop (Thermo Fisher, Wilmington, DE, USA) and copy number was calculated based on the mass of gBlock RNA.

### RNA extraction

Molecular grade water (Corning, Manassas, VA, USA), prepared stool, and serum matrix were spiked with IVT RNA standard and extracted using the Quick-DNA/RNA Viral MagBead Kit (Zymo, Irvine, CA, USA) and KingFisher Flex Purification System 96 PCR head (Thermo Fisher, Singapore). For the limit of detection determination and spiked serum experiments, 2 µL 10^0^–10^7^ copies/µL IVT RNA was spiked into 98 µL RNase-free water/pooled negative serum and then 100 µL 2× DNA/RNA Shield (Zymo, Irvine, CA, USA) were added to make a 200 µL sample in 96-Deep Well Plate (Cat#95040460, Thermo Fisher) for KingFisher Flex extraction. For testing of stool specimens spiked with synthetic filovirus RNA, 198 µL stool matrix was spiked with 2 µL 10^0^–10^7^ copies/µL IVT RNA to make 200 µL test samples. The final concentration of tested samples was 10^1^–10^8^ copies/mL. For the specificity test, 20 µL respiratory clinical specimens were mixed with 80 µL molecular water, followed by addition of 100 µL 2× DNA/RNA Shield for a final volume of 200 µL test sample. Using an extraction program with DNase digestion (R2140 Quick-DNARNA Viral Magbead wDNaseI v2.bdz; https://github.com/Zymo-Research/KingFisher.Flex.R2132.Script) provided by Zymo Research, the extracted RNA was eluted in 50 µL nuclease-free water.

### Reverse transcription and SYBR Green PCR

SuperScript IV First-Stand Synthesis System (Invitrogen, Carlsbad, CA, USA) was used for reverse transcription. 11.5 µL of RNA was mixed with 1 µL 50 µM random hexamer and 1 µL 10 mM dNTPs before heating at 65°C for 5 min. After cooling on ice for at least 1 min, a 6.5 µL mixture containing 1 µL 40 U/µL ribonuclease inhibitor, 4 µL 5X SSIV buffer, 1 µL 100 mM DTT, and 0.5 µL 200 U/µL SuperScript IV Reverse Transcriptase was added to make a final volume of 20 µL. Reactions were incubated at 25°C 10 min, 50°C 15 min, and 80°C 10 min. Then 1 µL RNaseH was added and incubated at 37°C for 20 min to digest RNA.

All qPCR reactions were performed using the QuantiNova SYBR Green PCR Kit (Qiagen, Germantown, MD, USA) and the 7500 Real-Time PCR System (Applied Biosystems, Waltham, MA, USA). Reactions were performed in a final volume of 20 µL containing 10 µL 2× SYBR Green PCR Master Mix, 2 µL of each 10 µM primer, 2 µL nuclease-free water, and 4 µL cDNA template. Reactions were incubated at 95°C 5 min, followed by 40 cycles of 94°C 15 s, 51°C 30 s, 72°C 60 s, followed by a melt curve stage at 94°C 15 s, 60°C 60 s, and 95°C 15 s (ramp rate of 1°C/s). Analysis was performed using 7500 Real-Time PCR System software version 2.0.6 (Applied Biosystems, Waltham, MA, USA). The amplicon was checked with FlashGel System (Lonza, Rockland, ME, USA). 1 µL 6× loading buffer (NEB, Ipswich, MA, USA) was added to 5 µL PCR product to perform electrophoresis on 1.2% FlashGel Cassettes (Cat#57023, Lonza, Rockland, ME, USA). For analytical sensitivity calculation, an in-house Rscript (https://github.com/greninger-lab/assay-validation/tree/main/LoD_analysis) was used for probit regression analysis. To construct standard curves for each of the 10 templates, Cts corresponding to 10^3^ to 10^8^ copies/reaction were used.

### Filovirus RNA preparation

Stocks of ten filovirus isolates (three Ebola virus isolates, two Marburg virus isolates, one Bundibugyo virus isolate, one Tai Forest virus isolate, one Reston virus isolate, one Sudan virus isolate, and one recombinant Lloviu virus) (25) used for accuracy testing and amplicon sequencing were generated in the BSL-4 facility of Boston University’s National Emerging Infectious Diseases Laboratories following approved standard operating procedures in compliance with local and national regulations pertaining to handling BSL-4 pathogens and Select Agents. With the exception of Lloviu virus, filovirus isolates were kindly provided by the NIH NIAID Rocky Mountain laboratories. Each of these viruses was propagated in Vero E6 cells in DMEM supplemented with 2 mM l-glutamine, 100 µg/mL Primocin, and 2% FBS. Virus titers were determined in Vero E6 cells by tissue culture infectious dose 50 (TCID_50_) assay using the Spearman and Kärber algorithm (25, 26). About 0.25 mL of filovirus stocks was inactivated in 0.75 mL TRIzol LS, according to approved SOPs and as previously described (27), before moving to BSL-2 for further experiments. Filovirus species and titers are listed in Table S3. About 400 µL filovirus-TRIzol LS was used for RNA extraction with Zymo Direct-zol RNA Purification Kits (Zymo Research Corp., Irvine, CA, USA). The extracted RNA is eluted in 50 µL RNase-free water, either used for cDNA synthesis or diluted with RNase-free water before cDNA synthesis for filovirus sensitivity test.

### Metagenomic and amplicon NGS and data analysis

Filovirus isolates were confirmed by metagenomic NGS. SuperScript IV First-Stand Synthesis System (Invitrogen, Carlsbad, CA, USA) was used for reverse transcription. About 19 µL of RNA was mixed with 1 µL 50 µM random hexamer before heating at 65°C for 5 min. After cooling on ice for at least 1 min, a 11 µL mixture containing 6 µL 5× SSIV buffer, 3 µL dNTP (10 mM), 1.5 µL 100 mM DTT, and 0.5 µL 200 U/µL SuperScript IV Reverse Transcriptase was added to make a final volume of 31 µL. Reactions were incubated at 23°C for 10 min , 50°C for 15 min, 94°C for 2 min, 10°C hold. Second strand cDNA was synthesized with Sequenase Version 2.0 DNA Polymerase (Thermo Fisher Scientific Baltics UAB, Vilnius, Lithuania) with 3.7 µL 5× Sequenase buffer 1.1 µL ddH2O and 0.225 µL Sequenase enzyme per reaction. Reactions were incubated at 10°C for 2 min, ramp to 37°C over 8 min: 15°C for 2 min, 20.5°C for 2 min, 26°C for 2 min, 31.5°C for 2 min, 37°C for 8 min, and hold at 10°C. The double-stranded cDNA was cleaned up with 1.8× AMPure beads (Beckman Coulter, Brea, CA, USA) following manufacturer instructions. The library was constructed with Illumina DNA Prep kit (Illumina, San Diego, CA, USA) and purified with 1× AMPure beads before loading to NovaSeq 6000 for sequencing.

Pan-filovirus PCR amplicons were purified with 1.0× AMPure beads and eluted with 25 µL molecular biology grade water. After quantifying with Qubit Fluorometer with Qubit dsDNA HS Assay Kits (Thermo Fisher Scientific, Eugene, OR, USA), amplicon was diluted down to 1 ng/µL. The library was constructed using Nextera XT DNA Library Prep Kit (Illumina, San Diego, CA, USA) following the manufacturer’s instructions with 16 cycles of amplification. Then the amplified library was purified with 1.0X AMPure beads again before loading to NovaSeq 6000 for sequencing.

Sequencing data were uploaded to CZID (https://czid.org) for analysis and identification (28). Non-host reads and candidate reference genomes generated by CZID were downloaded and inputted into Geneious Prime 2023.2.1 for further validation. Non-host reads were mapped to the candidate reference genomes using Geneious Mapper with default parameters. Candidate reference genome with the most mapped reads was recognized as the validated reference.

## RESULTS

### Phylogenetic analysis of filovirus and primer design

**Fig 1:**
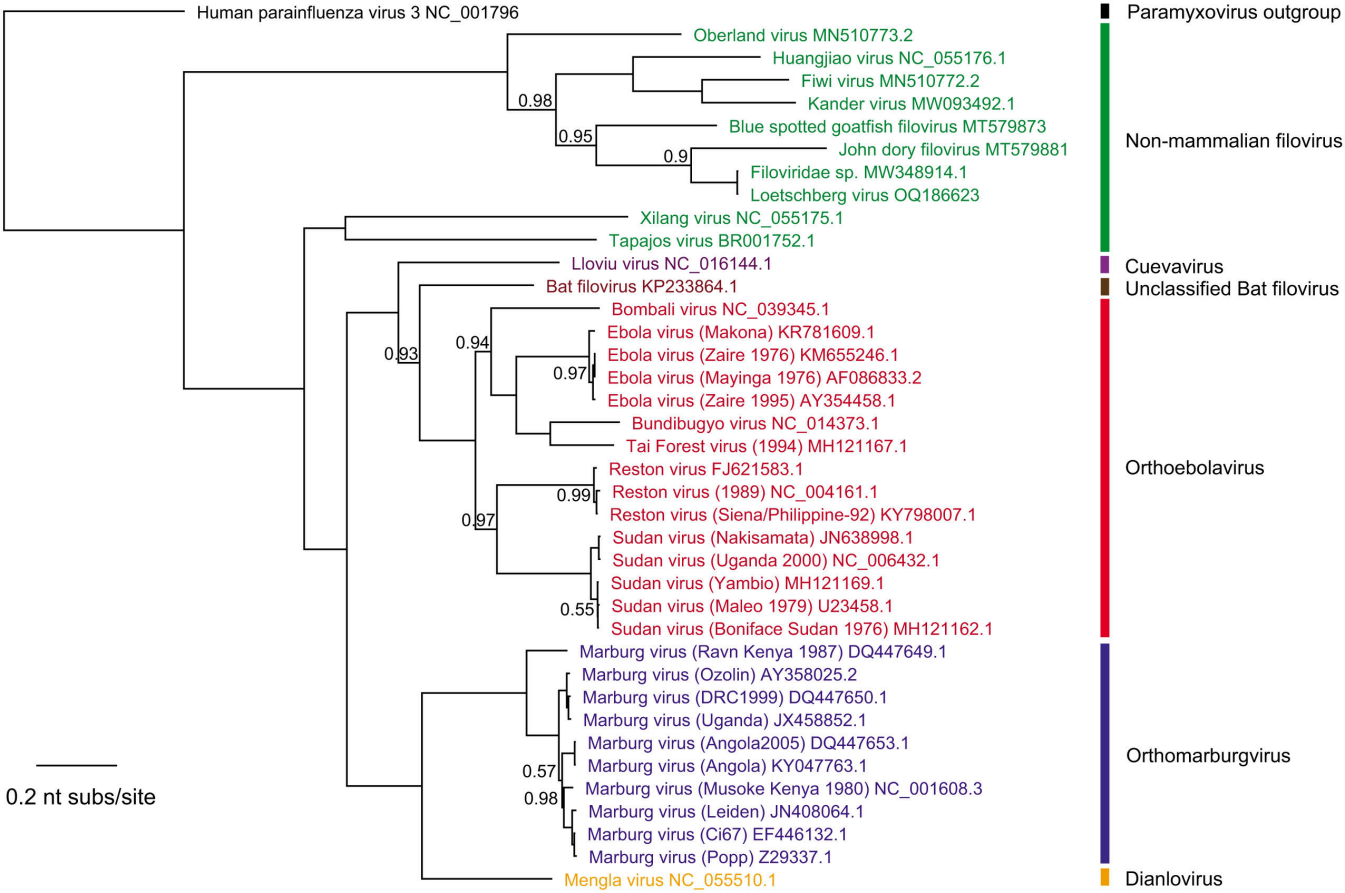
Phylogenetic tree of 38 filovirus L genes. A phylogenetic tree of 38 representative filoviruses L gene sequences, constructed using Clustal Omega/FastTree with 1,000 bootstrap replicates and using human parainfluenza virus 3 as outgroup, is depicted. The four non-mammalian filovirus genera are shown in green (*Oblavirus*, *Striavirus*, *Tapjovirus*, and *Thamnovirus*). Mammalian filovirus genera are shown in purple (*Cuevavirus*), red (*Orthoebolavirus*), blue (*Otthomarburgvirus*), and orange (*Dianlovirus*). An unclassified bat filovirus sequence is shown in brown. The scale bar shows the distance in the unit of nucleotide substitutions per site. Nodes with bootstrap support values less than 1 are labeled with the support value.

**Fig 2:**
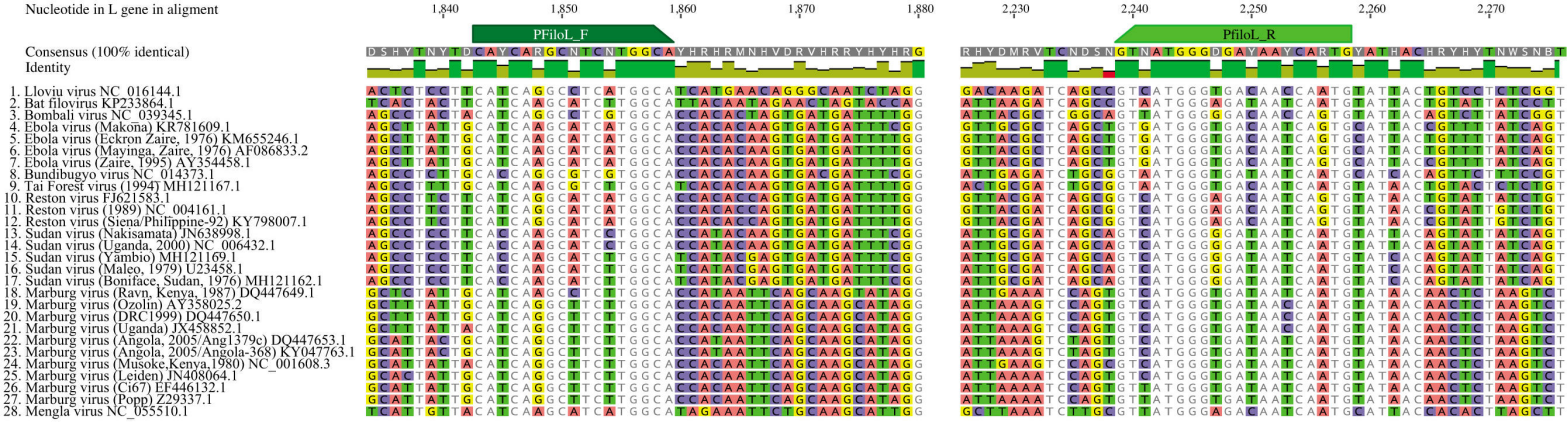
Multiple alignment of the 28 mammalian filovirus L genes with nucleotide sequences and positions for the pan-filovirus primers. An alignment of 28 mammalian filovirus L genes from Clustal Omega v1.2.3 and 100% identity consensus sequence are shown. The locus targeted by the forward and reverse primers designed in this study are marked in dark and light green.

To design initial PCR primers, 37 full and partial filovirus L gene sequences from all eight genera were downloaded from NCBI GenBank and aligned with ClustalOmega v1.2.3. Nine sequences of four non-mammalian genera (*Oblavirus*, *Striavirus*, *Tapjovirus*, and *Thamnovirus*) were grouped into one cluster (Fig. 1). The other 28 sequences belong to the four mammalian genera (*Cuevavirus*, *Dianlovirus*, *Orthomarburgvirus*, and *Orthoebolavirus*) and one unclassified bat-borne partial filovirus genome (Bat filovirus isolate BtFV/DH04, KP233864.1). Given the long phylogenetic distance between mammalian and non-mammalian filoviruses and the incomplete L gene sequences of some non-mammalian filoviruses, we focused PCR primer design on the 28 mammalian filovirus sequences. The accession number and sequence of 28 reference filovirus are listed in File S1. The degenerate primers designed match 100% to all 28 reference sequences with a 64-fold degeneracy for the forward primer and 96-fold for the reverse primer (Fig. 2).

### *In silico* accuracy test of pan-filovirus primers

To test the *in silico* accuracy of our pan-filovirus primers on a larger array of filovirus sequences, we downloaded 3,051 filovirus L gene sequences from NCBI Nucleotide with complete sequence in the binding regions of our primer sets (Table S1). About 3,030 out of 3,051 (99.31%) filovirus L sequences matched perfectly with our pan-filovirus degenerate primers. When allowing one nucleotide mismatch, 3,040 out of 3,051 (99.64%) filovirus L sequences matched. To compare our primers with previously published sets (21, 23, 24), we performed the same *in silico* test with the published primers and found our primers had better coverage than previously published L primers and similar coverage as previously published NP-targeting pan-filovirus primers (Table 1) (24).

**TABLE 1:** Digital accuracy test of pan-filovirus primer on existing filovirus sequences^*a*^

	Pan-filovirus L primers	L primers (21, 21)	L primers (23)	NP primers (24, 24)
Tested sequence number	3,051	3,051	3,051	3,183
Minimum length of template (bp)	2,682	2,682	2,682	745
Average length of template (bp)	6,648	6,648	6,648	2,209
Maximum length of template (bp)	6,996	6,996	6,996	2,250
Perfect sequence matches (#)	3,030	11	2,851	3,156
Perfect sequence matches (%)	99.31%	0.36%	93.44%	99.15%
Sequences with one mismatch (#)	3,040	900	2,956	3,156
Sequences with one mismatch (%)	99.64%	29.50%	96.89%	99.15%

^*a*^ Tested sequence number: the number of L/NP genes used for each test. Minimum/Average/Maximum length of template: the length of L/NP gene sequence used for each test. Perfect sequence matches (#): the number of L/NP gene sequence that perfectly matched a primer set. Perfect sequence matches (%): the percentage of L/NP gene sequences that perfectly matched a primer set. Sequences with one mismatch (#): the number of L/NP gene sequences that matched a primer set with one mismatch. Sequences with one mismatch (%): the percentage of L/NP gene sequences that matched a primer set with one mismatch.

### Specificity

The specificity of the pan-filovirus RT-PCR assay was confirmed by testing 20 remnant clinical specimens positive for ten different non-filovirus species: human parainfluenza virus 1 (HPIV1), human parainfluenza virus 2 (HPIV2), human parainfluenza virus 3 (HPIV3), human parainfluenza virus 4 (HPIV4), human metapneumovirus (hMPV), adenovirus (AdV), respiratory syncytial virus (RSV), Nipah virus (NiV), Rhinovirus C (RhVC), or human bocavirus (BoV) (Table S2). In each case, the pan-filovirus RT-PCR assay result was negative. The negative results were also confirmed by agarose gel electrophoresis analysis of the RT-PCR reactions demonstrating no amplicon generated in these reactions (Fig. S1).

### Analytical sensitivity

**Fig 3:**
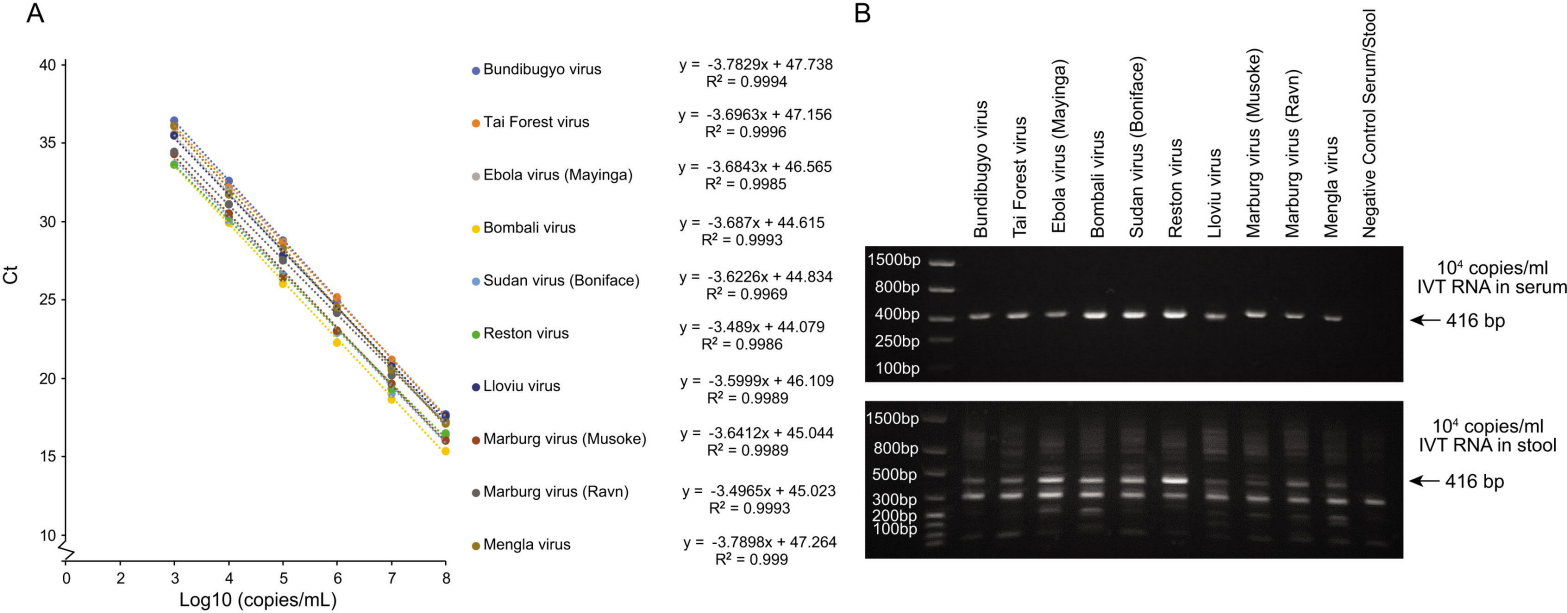
Limit of detection of pan-filovirus RT-PCR assay. (**A**) Standard curves for serially diluted (10^3^–10^8^ copies/mL) standard templates. Average Ct value of eight replicates in each dilution was used for the regression analysis. (**B**) Agarose gel electrophoresis of PCR product from the 10^4^ copies/mL standard template in negative serum or stool matrix.

To determine the analytical sensitivity of the pan-filovirus RT-PCR, we ordered synthetic DNA from 10 different mammalian filoviruses and created *in vitro* transcribed RNA. The RNA templates were diluted in nuclease-free water from 10^1^ to 10^8^ copies/mL per sample for extraction. For each template, each dilution was tested with eight replicates, and probit regression analysis was performed to determine the limit of detection. All 10 standard templates were detected by pan-filovirus assay with excellent linearity (Fig. 3A; Fig. S2). After probit analysis, the analytical sensitivity of pan-filovirus assay for 10 standard IVT templates ranged from 178 copies/mL (8.2 copies/reaction) for Ebola virus and Sudan virus to 3,354 copies/mL (154.3 copies/reaction) for Marburg virus (Musoke) (Table 2). The original qPCR results are listed in Table S4.

**TABLE 2:** Analytical sensitivity of pan-filovirus assay on 10 synthetic filovirus sequences

	Limit of detection (copies/mL)	Limit of detection (copies/reaction)
Bundibugyo virus	426	19.6
Tai Forest virus	212	9.8
Ebola virus (Mayinga)	178	8.2
Bombali virus	195	9.0
Sudan virus (Boniface)	178	8.2
Reston virus	234	10.8
Lloviu virus	212	9.8
Marburg virus (Musoke)	3,354	154.3
Marburg virus (Ravn)	1,798	82.7
Mengla virus	1,161	53.4

To confirm the sensitivity of the pan-filovirus assay in specimen matrices of differing complexity, we spiked IVT RNA templates into clinical remnant human serum and animal stool samples. Adding mammalian specimen matrix increased the background of RT-PCR, especially with the more complex animal stool matrix. However, the target band was still detectable at 10^4^ copies/mL (460 copies/reaction) for all 10 standard templates in both matrices (Fig. 3B).

### Accuracy and sensitivity test on filovirus genomic RNA

**Fig 4:**
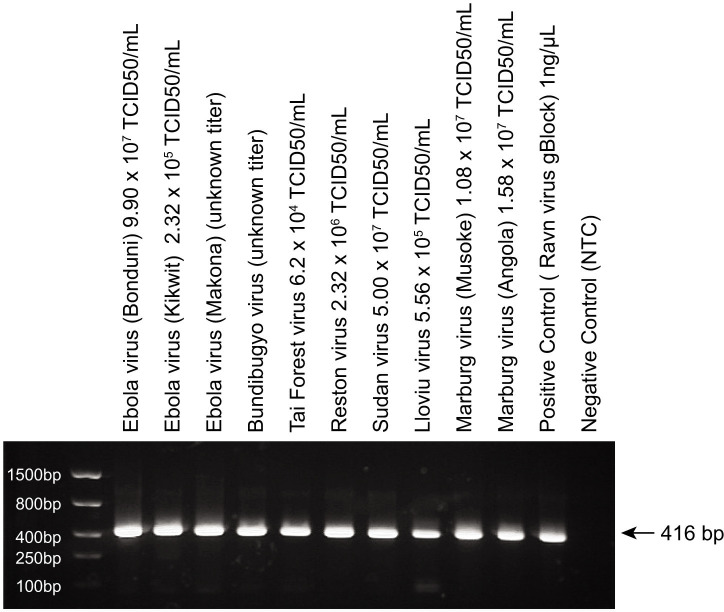
Agarose gel electrophoresis of pan-filovirus PCR assay on 10 genomic RNAs from filovirus isolates. Positive control is 1 ng/µL Ravn virus gBlock. Negative control is non-template control (NTC).

The identities of 10 filovirus isolates were first confirmed by metagenomic next-generation sequencing (Table S3). As shown in Fig. 4, specific, strong amplicon bands were detected by pan-filovirus PCR assay in all 10 filovirus samples, matching the accuracy seen on synthetic filovirus sequence. The original qPCR result is listed in Table S5. PCR amplicons from the filovirus isolates were purified, tagmented, and deep sequenced and reads were uploaded to CZID for candidate reference genome sequence identification (Table 3). The species identified by CZID for the pan-filovirus PCR amplicon matched the known species information of all 10 isolates with 100% identity in the amplicon region (Table S3; Fig. S3).

**TABLE 3:** Amplicon NGS and species identification of genomic RNA from 10 filovirus isolates

Sample name	Species identified by CZID	Top reference sequence in CZID^a^	Annotation
Ebola virus (Bonduni)	Zaire ebolavirus(*Orthoebolavirus zairense*)	KC242791.1	Zaire ebolavirus isolate EBOV/H.sapiens-tc/COD/1977/Bonduni, complete genome
Ebola virus (Kikwit)	Zaire ebolavirus(*Orthoebolavirus zairense*)	KU321102.1	Zaire ebolavirus isolate Ebola virus/H.sapiens-tc/COD/1995/Kikwit-9510621–1259D7 membrane-associated protein VP24 (VP24) gene, partial cds; and polymerase (L) gene, complete cds
Ebola virus (Makona)	Zaire ebolavirus(*Orthoebolavirus zairense*)	KR653294.1	Zaire ebolavirus isolate Ebola virus/H.sapiens-wt/SLE/2014/Makona-20140174, partial genome
Bundibugyo virus	Bundibugyo ebolavirus(*Orthoebolavirus bundibugyoense*)	NC_014373.1	Bundibugyo ebolavirus, complete genome
Tai Forest virus	Tai Forest ebolavirus(*Orthoebolavirus taiense*)	MH121167.1	Tai Forest ebolavirus isolate Ebola virus/H.sapiens-tc/Cote d'Ivoire/1994/Tai Forest-R4371, complete genome
Reston virus	Reston ebolavirus(*Orthoebolavirus restonense*)	KY798005.1	Reston ebolavirus isolate USA_VA_1989(811952), complete genome
Sudan virus	Sudan ebolavirus(*Orthoebolavirus sudanense*)	KY425644.1	Sudan ebolavirus isolate IRF0154, partial genome
Lloviu virus	Lloviu cuevavirus(*Cuevavirus lloviuense*)	JF828358.1	Lloviu virus strain MS-Liver-86/2003, complete genome
Marburg virus (Musoke)	Marburg Marburgvirus(*Orthomarburgvirus marburgense*)	AY430366.1	Lake Victoria marburgvirus strain pp4 guinea pig nonlethal variant, complete genome
Marburg virus (Angola)	Marburg Marburgvirus(*Orthomarburgvirus marburgense*)	DQ447657.1	Lake Victoria marburgvirus - Angola2005 strain Ang0214, complete genome

^a^ Top reference sequence in CZID is the first recommended NCBI reference in the list by CZID.

For sensitivity test on full-length filovirus genomic RNA, eight inactivated filovirus culture isolates with known TCID_50_/mL titer [Ebola virus (Bonduni), 9.90 × 10^7^; Ebola virus (Kikwit), 2.32 × 10^5^; Tai Forest virus, 6.20 × 10^4^; Reston virus, 2.32 × 10^6^; Sudan virus, 5.00 × 10^7^; Lloviu virus, 5.56 × 10^5^; Marburg virus (Musoke), 1.08 × 10^7^; and Marburg virus (Angola), 1.58 × 10^7^] were diluted across ten 10-fold dilutions and eight replicates of the pan-filovirus PCR assay were run at each dilution. After probit analysis, we obtained the 95% limit of detection for each virus in TCID_50_/mL (Table 4). The assay was most sensitive to Tai Forest virus (0.012 TCID_50_/mL) and least sensitive to Sudan virus (44.17 TCID_50_/mL).

**TABLE 4:** Analytical sensitivity of pan-filovirus assay on genomic RNA from 10 filovirus isolates

	TCID_50_/mL
Ebola virus (Bonduni)	20.50
Ebola virus (Kikwit)	1.06
Tai Forest virus	0.01
Reston virus	0.99
Sudan virus	44.17
Lloviu virus	13.88
Marburg virus (Musoke)	15.84
Marburg virus (Angola)	3.58

## DISCUSSION

The last decade has witnessed a surge in the frequency and intensity of outbreaks caused by filoviruses, a family of viruses notorious for their high mortality rates and potential to cause public health crises. Over the last 5 years, outbreaks of filoviruses have been reported in multiple regions, challenging the resilience of public health systems globally. Notable incidents include the Ebola virus outbreak in the Democratic Republic of Congo (DRC) in 2018–2020 and the Marburg virus outbreak in Uganda in 2022 (29, 30). In recent years, viruses derived from zoonotic origins have gained enormous attention due to the potential for these cross-overs to lead to serious diseases in humans (31). For example, bats have been implicated as reservoirs for a number of zoonotic viruses of public health concern, including filoviruses (26, 27, 32, 33). There have been some effective filovirus detection methods published so far, either for clinical diagnosis or for research (34, 35). However, current filovirus detection methods in zoonosis face several challenges, like sensitivity and specificity limitations, single-pathogen focus, and inability to detect newly emerging filovirus strains (15, 16, 24). To address these challenges, there is a need to develop new pan-filovirus RT-PCR assays capable of detecting a broad range of filovirus variants in a broad range of potential specimens.

The pan-filovirus RT-PCR assay detailed in this study successfully identified representatives from all four mammalian filovirus genera. This assay exhibits a remarkable detection capability, as it can identify over 99% of presently documented mammalian filovirus sequences. In response to the observed diversity, a singular primer pair, incorporating multiple degenerate bases, was employed, yielding an analytical sensitivity ranging from 8.2 to 154.3 copies per reaction. This performance is comparable to that of another pan-filovirus SYBR Green RT-PCR assay targeting the NP gene (24). Moreover, the implementation of a two-step RT-PCR strategy enhances viral screening and discovery by allowing targeting of other viral families using the same cDNA material.

Beyond its specificity and sensitivity, the 416 bp amplicon generated by the pan-filovirus RT-PCR assay is sufficiently long for accurate species identification. This represents the longest amplicon for a pan-filovirus RT-PCR assay described to date (15, 16, 18–24). While the extended amplicon may affect sensitivity in degraded samples, it increases the amount of sequence available for species identification and the detection of novel filoviruses. Integration with amplicon NGS facilitates identification with minimal PCR product and ability to pool screening PCRs from a high number of specimens.

As with any pan-family RT-PCR reaction, our assay has limitations. By targeting a highly conserved region within the filovirus genome, the current resolution of the assay is at the level of viral species. Many isolates share identical sequences within the amplicon region and additional sequencing may be required for subspecies-level identification. Targeting the L gene may reduce sensitivity compared to other RT-PCR assays given the transcriptional gradient often seen in negative-stranded RNA viruses. Furthermore, we have not yet used the assay to discover any new filovirus species, although we note our primers perfectly match the sequence of the newly discovered Dehong virus (OP924273.1) which was reported after the primers were designed.

Our newly devised pan-filovirus RT-PCR assay offers a cost-effective and efficient means of identifying mammalian filoviruses in a variety of specimens. When coupled with amplicon NGS, this assay facilitates the identification of existing filoviruses and detection of previously unknown filoviruses with minimal sample volume. The advancement of surveillance techniques for filoviruses in diverse human, animal, and environmental samples is crucial for fortifying our preparedness for prospective outbreaks of filovirus hemorrhagic fever.

## Data Availability

Sequencing data were uploaded to NCBI SRA under BioProject PRJNA1095911.

## SUPPLEMENTAL MATERIAL

Figure S1 spectrum.01010-24-s0001.pdf Specificity test of pan-filovirus RT-PCR assay.

Figure S2 spectrum.01010-24-s0002.pdf Gel electrophoresis of standard templates.

Figure S3 spectrum.01010-24-s0003.pdf Pairwise alignment of amplicon NGS.

Supplemental file S1 spectrum.01010-24-s0004.txt Fasta alignment of 28 L gene sequences used in primer design.

Supplemental file S2 spectrum.01010-24-s0005.txt Synthetic L gene fragments.

Supplemental tables spectrum.01010-24-s0006.xlsx Tables S1-S5.

ASM does not own the copyrights to Supplemental Material that may be linked to, or accessed through, an article. The authors have granted ASM a non-exclusive, world-wide license to publish the Supplemental Material files. Please contact the corresponding author directly for reuse.
