# Supplementary material for: A high-throughput, polymerase-targeted RT-PCR for broad detection of mammalian filoviruses
Source: Microbiol Spectr. 2024 Jul 24;12(9):e01010-24. doi: 10.1128/spectrum.01010-24 (PMC11370238; doi:10.1128/spectrum.01010-24)
Supplement: Figure S1 — Specificity test of pan-filovirus RT-PCR assay. [file spectrum.01010-24-s0001.pdf]

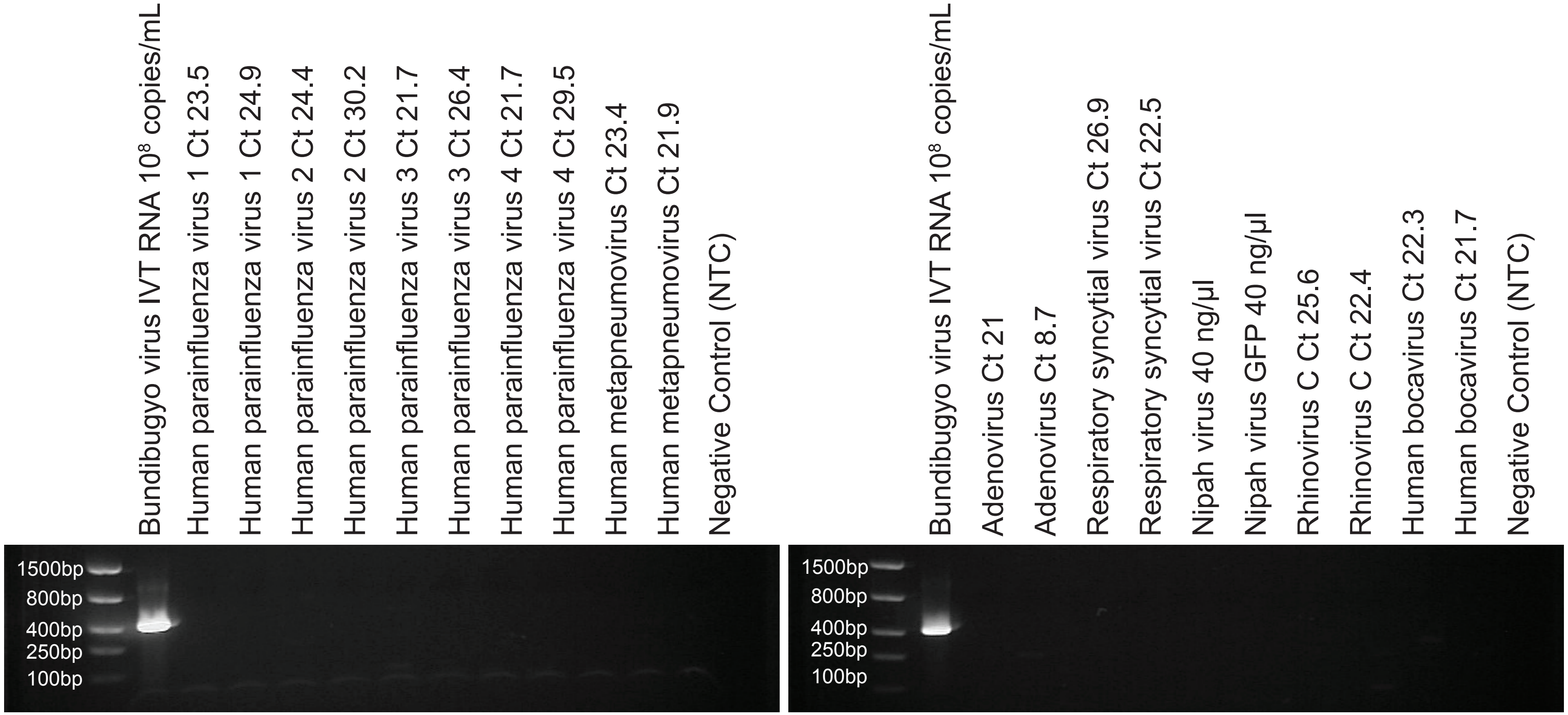

Figure S1. Specificity test of pan-filovirus RT-PCR assay.
Twenty samples from ten species of viruses (HPIV1, HPIV2, HPIV3, HPIV4, hMPV, AdV, RSV, NiV, RhVC, BoV) were used for specificity test.
The Ct value of each virus sample is identified by primers specific for the targeted virus. The concentration of Nipah virus RNA is noted.
Bundibugyo virus IVT RNA 10^8 copies/ml (9.2×10^5 copies/reaction) was used as a positive control. Negative PCR control is non-template control (NTC).
