# Supplementary material for: A high-throughput, polymerase-targeted RT-PCR for broad detection of mammalian filoviruses
Source: Microbiol Spectr. 2024 Jul 24;12(9):e01010-24. doi: 10.1128/spectrum.01010-24 (PMC11370238; doi:10.1128/spectrum.01010-24)
Supplement: Figure S2 — Gel electrophoresis of standard templates. [file spectrum.01010-24-s0002.pdf]

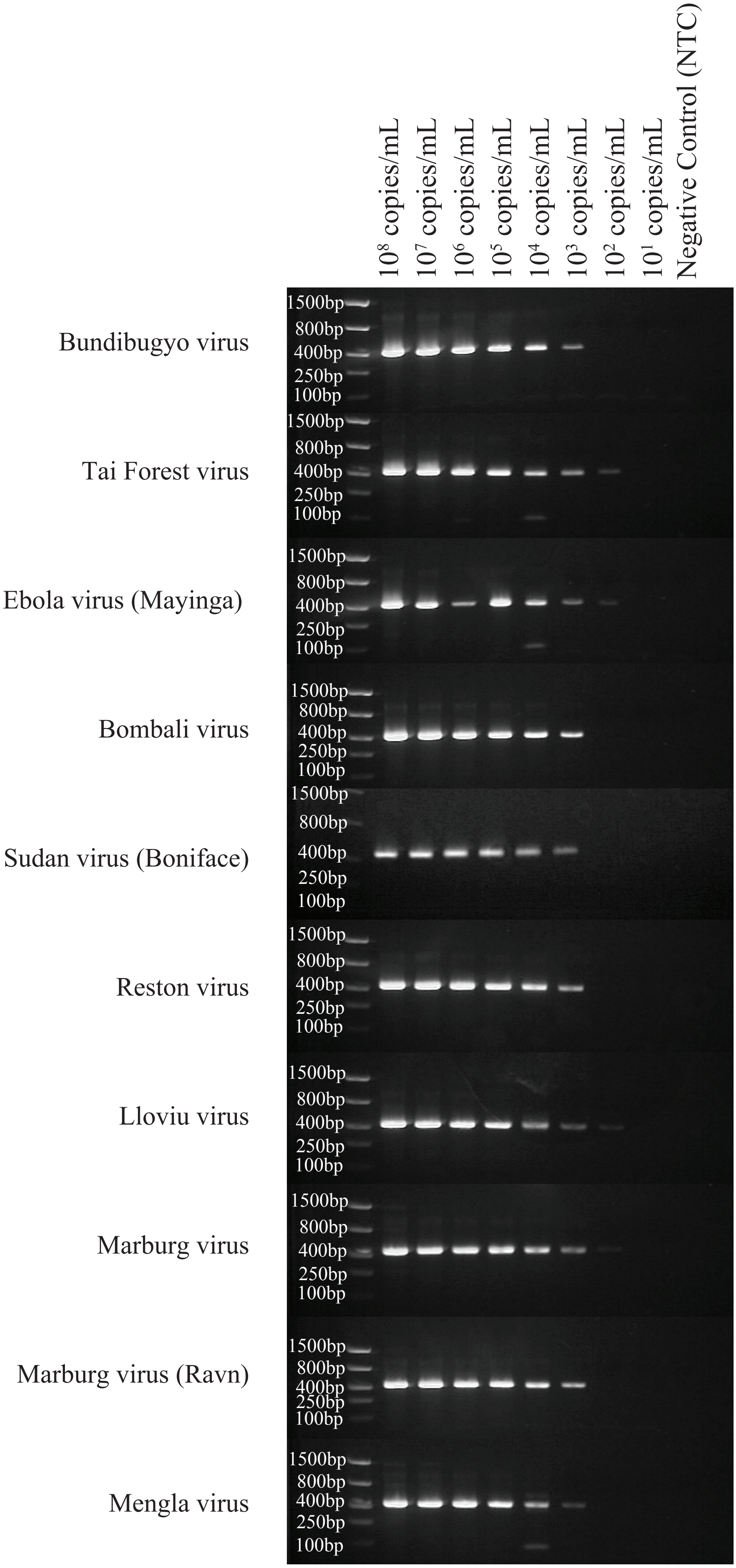

Figure S2. Agarose gel electrophoresis of serially diluted standard templates (10^8 to 10^1 copies/mL). Standard IVT RNA were serially diluted ten-fold into molecular grade water for pan-filovirus RT-PCR assay. Negative control is a non-template PCR control (NTC).
