# Supplementary material for: A high-throughput, polymerase-targeted RT-PCR for broad detection of mammalian filoviruses
Source: Microbiol Spectr. 2024 Jul 24;12(9):e01010-24. doi: 10.1128/spectrum.01010-24 (PMC11370238; doi:10.1128/spectrum.01010-24)
Supplement: Figure S3 — Pairwise alignment of amplicon NGS. [file spectrum.01010-24-s0003.pdf]

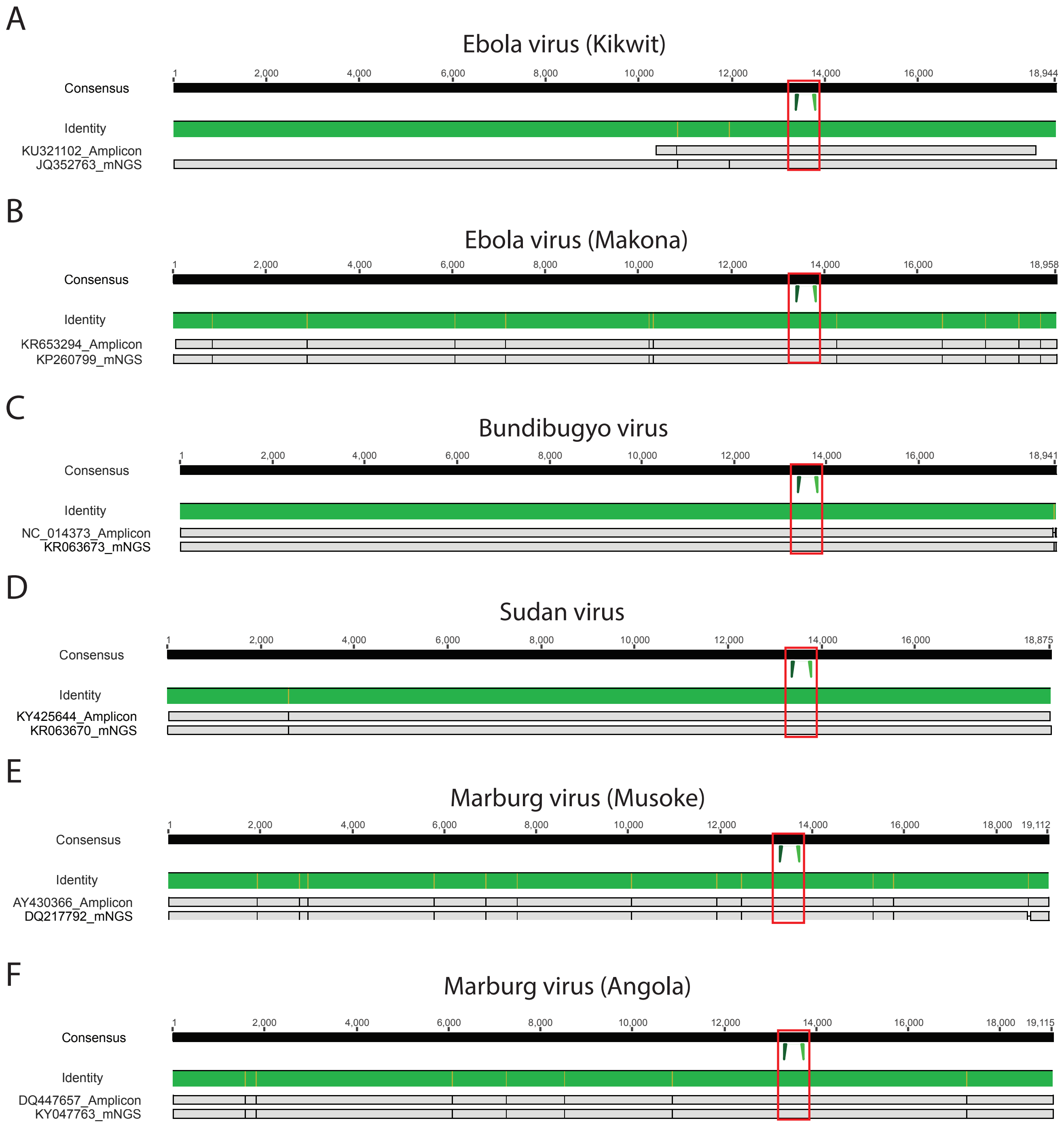

Figure S3. Pairwise alignment of amplicon NGS and mNGS reference genomes identified by CZID. Green triangles indicate the pan-filovirus forward and reverse primers designed in this study. Red rectangle indicates the 100% identical region where primers targeted.
